# REDA: A New Methodology to Validate Sensor Systems for Person Detection under Variable Environmental Conditions

**DOI:** 10.3390/s22155745

**Published:** 2022-08-01

**Authors:** Christian Meltebrink, Magnus Komesker, Carolina Kelsch, Daniel König, Mario Jenz, Marvin Strotdresch, Benjamin Wegmann, Cornelia Weltzien, Arno Ruckelshausen

**Affiliations:** 1 Faculty of Engineering and Computer Science, University of Applied Sciences Osnabrück, 49076 Osnabrück, Germany; magnus.komesker@hs-osnabrueck.de (M.K.); ckelsch@edu.unisinos.br (C.K.); philipp-daniel.koenig@hs-osnabrueck.de (D.K.); mario.jenz@hs-osnabrueck.de (M.J.); marvin.strotdresch@hs-osnabrueck.de (M.S.); a.ruckelshausen@hs-osnabrueck.de (A.R.); 2 Agromechatronic, Technische Universität Berlin, 10623 Berlin, Germany; cornelia.weltzien@tu-berlin.de; 3 B. Strautmann & Söhne GmbH u. Co. KG, 49196 Bad Laer, Germany; b.wegmann@strautmann.com; 4 Faculty of Electronic Engineering, Unisinos University, São Leopoldo 93022-750, Brazil; 5 Leibniz Institute for Agricultural Engineering and Bioeconomy (ATB), 14469 Potsdam, Germany

**Keywords:** safety of autonomous machines, object detection systems, perception systems, image based sensor systems

## Abstract

Perception of the environment by sensor systems in variable environmental conditions is very complex due to the interference influences. In the field of autonomous machines or autonomous vehicles, environmental conditions play a decisive role in safe person detection. A uniform test and validation method can support the manufacturers of sensor systems during development and simultaneously provide proof of functionality. The authors have developed a concept of a novel test method, “REDA”, for this purpose. In this article, the concept is applied and measurement data are presented. The results show the versatile potential of this test method, through the manifold interpretation options of the measurement data. Using this method, the strengths and weaknesses of sensor systems have been identified with an unprecedented level of detail, flexibility, and variance to test and compare the detection capability of sensor systems. The comparison was possible regardless of the measuring principle of the sensor system used. Sensor systems have been tested and compared with each other with regard to the influence of environmental conditions themselves. The first results presented highlight the potential of the new test method. For future applications, the test method offers possibilities to test and compare manifold sensing principles, sensor system parameters, or evaluation algorithms, including, e.g., artificial intelligence.

## 1. Introduction

With the development of autonomous machines, the perception of the environment and the associated safe detection of people are the enablers for these machines. For example, in agriculture, in parallel with the functionality of autonomous machines, the safe operation and perception of the environment is also considered from the beginning [1].

One variant for safeguarding autonomous machines is the use of supervisors who can observe the autonomous operation, intervene if necessary, and prevent dangerous situations. Various warning systems were tested in a study [2]. If these systems are to manage without a supervisor, the selection of sensor systems and the corresponding evaluation method is of decisive importance. Ignatious et al. present an overview of different sensor systems for autonomous vehicles [3]. In addition, Rosique et al. introduce the different measurement principles of the different perception systems, quantify them by features, and show elements that are important in a model-based development [4].

Safe environment perception also plays an essential role in the field of autonomous driving. Hoss et al. conduct a literature review on this subject and come to the conclusion that there is a lack of test methods that can prove the reliability of perception systems [5]. Skruch et al. define a measurement standard that can be used to evaluate the large number of safety-critical scenarios in road traffic and consequently reduce the number of scenarios to be tested [6].

For safe environment perception, it can be seen that classic functional safety and, for example, the standard series ISO 26262 [7], ISO 13849 [8], or ISO 25119 [9] has reached its limits. A sensor system that is safe from a functional safety point of view can still be unsafe and not suitable for environment perception. This is because the suitability of the sensor system must be validated with regard to various parameters, such as susceptibility to interference from environmental conditions in the area of use or the ability to detect a person. From the sensor manufacturer’s point of view, IEC TS 62998-1:2019-05 [10] defines development specifications to cover this part. In the automotive sector, the ISO/PAS 21448:2019-01 [11] standard provides a solution with the term SOTIF. In the area of mobile machinery, and in particular agricultural machinery, there are no normative specifications as yet.

**Figure 1:**
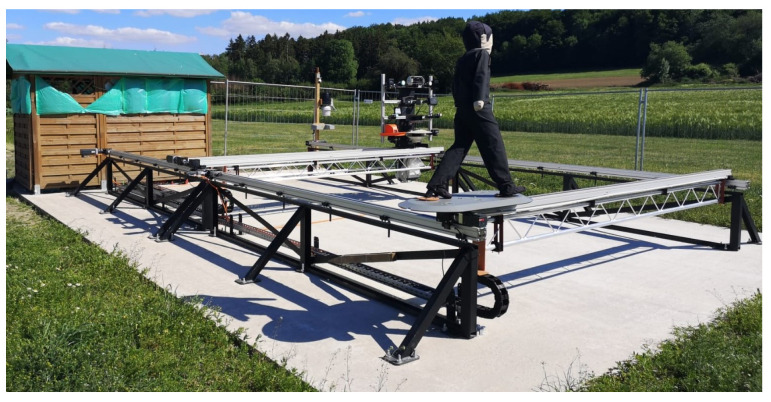
The first outdoor sensor test stand in agriculture is presented. The test stand can create REDAs and is working 24 h a day 365 days a year. Reprinted from Ref. [14].

In all these efforts to ensure safe operation of autonomous machines and vehicles, the safe environment perception will be the key enabler in bringing these machines to the market. The safe detection of persons in the different and challenging environmental conditions at the locations where the autonomous machines are used is the main focus. There should be a method that allows an independent assessment of the environment perception of autonomous machines. Specific tests for the sensor systems should combine and test these environmental conditions to evaluate the safe functionality. Kim et al. show how fog influences the perception of the environment and thus the functional safety of autonomous systems [12]. Hasirlioglu et al. present a test methodology for testing the rain influences of the sensor system [13]. They test automotive surround sensors with rain simulation. The authors present a method by which it is possible to evaluate different sensor systems with respect to their robustness against disturbances directly in the working environment and the reliable detection of persons in such environmental conditions [14]. This method is the enabler of autonomous systems because it is the final step in the development of autonomous systems. After a successful development of an entire autonomous system, this method can be used to prove the safe function of the perception systems. This method also makes it possible to compare and evaluate sensor systems in terms of detection capability and sensitivity to interference from environmental conditions in an unprecedented level of detail. As motivation and one example for an autonomous machine, an autonomous feeding mixer of the company B. Strautmann & Söhne GmbH u. Co. KG [1] has clearly shown the application of the new test method. During development, the need for non-contact sensor systems for the safe detection of people arose there, which could not be met by the market. With the novel test method, sensor systems are tested at the University of Applied Science Osnabrück (Germany) in cooperation with the company and the TÜV on the first outdoor sensor test stand in agriculture in the research project “Agro-Safety”, founded by the BMBF and B. Strautmann & Söhne GmbH u. Co. KG. In Figure 1 this test stand is shown.

Due to the complexity and diversity of environmental conditions, more and more use is being made of artificial intelligence in the interpretation of sensor data. According to Jiang et al., deep learning algorithms are promising approaches for fully autonomous vehicles [15]. Likewise, in the use of artificial intelligence, the progressive power of the software must be taken into account in order to generate safe artificial intelligence [16]. This work (the project “Agro-Saftey”) has inspired a larger test field with higher speeds for field-based crop production [17].

### 1.1. Concept of REDA and REDAM

For a better understanding of the article, the concept of the novel test method presented in [14] is summarized in the following lines. The test method is used to test sensor systems such as object detection systems (ODS). These systems consist of a sensor and an evaluation unit. The sensor scans the environment with its special measurement principle (e.g., LiDAR or RADAR) and the evaluation unit interprets the raw data. Before the interpretation of the raw data, different parameters can be set for the evaluation unit. As examples, different filters or multiple evaluations can be addressed. This can be used to make certain parametrizations of the sensor system. For the interpretation of the raw data, application-specific software is used. In the case of ODS, the evaluation unit would recognize objects based on the pre-processed raw data.

**Figure 2:**
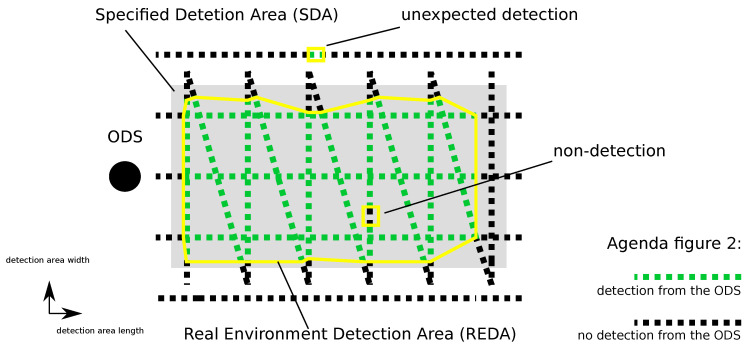
This figure shows the creation of a real environment detection area (REDA, yellow lines). Reprinted from Ref. [14].

To evaluate the influences of the environmental conditions on an ODS, the specified detection area (SDA) or region of interest of these systems is tested. This area is set in the sensor systems. Within this area, a system has to provide a reliable detection of objects. The ODS changes its output as soon as it has detected an object within the SDA. The test method used real environment detection areas (REDAs) to describe the SDA of a specific sensor with their parameters and algorithms in specific environmental conditions. Comparing the set SDA with the recorded REDA, a evaluation of the detection capability of specific test objects is possible. In Figure 2, the creation of a REDA and the comparability with the SDA of an ODS is shown.

An ODS is shown as a black dot in Figure 2. The SDA of the ODS is represented by a gray area. A test target is moved through the SDA from different directions. The relative position of the test target to the ODS is represented by small black and green square measuring points. The stringing together of these points results in the travel path of the test target relative to the ODS. These measuring points are stored with a defined period time. With the use of the test concept presented in this article, a test point is recorded every six milliseconds. The status at the ODS interface is stored for each test point. As already described, the ODS informs via the interface whether an object is inside its SDA or not. This status of the sensor is displayed by means of a green coloring (an object was detected in the SDA) and black coloring (no object was detected in the SDA) of the measuring points. At this point it should be noted that this marking of the measuring points is created independently of the position of the set SDA. The set SDA is only inserted subsequently for comparison and interpretation of the measuring points. Thus, green measuring points can also be recorded outside the gray SDA by this procedure. If the test target is outside the set SDA and the ODS nevertheless signals a detection, the measuring point for this relative position is marked with a detection (green). A REDA is now created only by the area of green measuring points. Thus, the actual detection area of the ODS at the current environmental conditions of the test can be described by the REDA with respect to the detection capability of test target by the ODS. By comparing the green and black measurement points with the SDA of the ODS, unexpected detections (green measurement point outside the SDA) and non-detections (black measurement point inside the SDA) can also be recorded.

**Figure 3:**
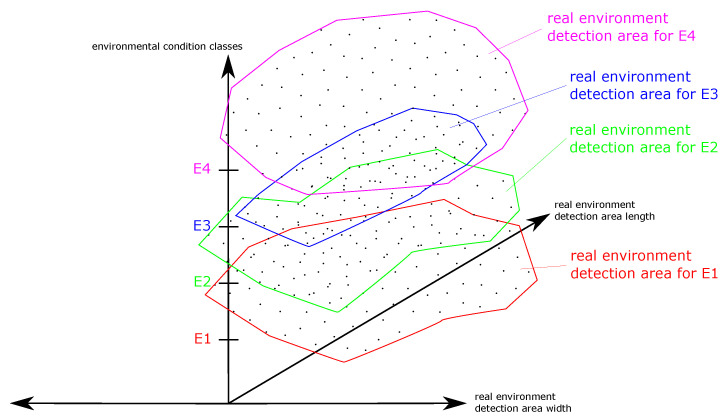
For illustration, in this figure a fictitious REDAM is shown. Reprinted from Ref. [14].

Different sensor systems have different strengths and weaknesses. In order to achieve the best possible results for a specific application of a sensor system, fusions of sensor systems are also a possible solution. A new very powerful tool for the development of such sensor fusions has already been described by the authors in [14]. Using the real environment detection area matrix (REDAM), a comparison of several REDAs becomes possible and thus the identification of weaknesses and strengths of individual sensor systems, their parameters, or algorithms used. For illustration, in Figure 3, a fictitious REDAM from [14] is shown.

In the REDAM of Figure 3, a REDA is applied to the abscissa axis and the ordinate axis. On the application axis, different environmental condition classes are plotted. Thus, a three-dimensional overview of different REDAs for different environmental condition classes is created. In this context, an environmental condition class is a specific environmental condition or a real combination of different environmental conditions in one class. In this way, individual environmental condition classes are compared with each other in a simple way for a sensor system. Additionally, a REDAM can be used to compare different sensor systems for a special environmental condition. The comparison of different parameters or algorithms of a sensor system is also comparable.

### 1.2. Objectives of Work

This article is intended to illustrate the power of the REDA test method. It can be used to identify the strengths and weaknesses of sensor systems with an unprecedented level of detail, flexibility, and variance as well as to test and compare the detection capability of sensor systems. It is shown that this test method is so powerful that it represents a solution—it is the enabler of autonomous systems. For this reason, the aim of this article is the application of the presented concept of the novel test method “REDA” [14]. Measurement data are presented and the resulting potential is shown by the versatile interpretation of the measurement data. The focus is on the interpretation of the data, which leads to different methods developed for the evaluation and analysis of the measurement data generated by this test concept.

## 2. Method and Measurements

This section describes the application of the new concept of the test method “REDA” described in Section 1.1 by the dynamic test stand shown in Figure 1. The test method and test stand has already been developed by the authors in the research project “Agro-Safety” [14]. In this project, the first use case of this method is an autonomous feeding mixer [1]. Based on the parameters of the use case, the first sensor test stand in the agricultural sector is realized. Because of the flexibility of the test method “REDA”, this method is usable for other use cases, such as for autonomous machines with higher speeds or autonomous vehicles.

Because the method and the test stand are already known as state of the art and the focus of this article is on data interpretation, this section deliberately summarizes the method and the measurements as an overview.

The “Agro-Safety” research project has met with great interest from eight different manufacturers of sensor systems from industry and the automotive sector. They are taking part in the tests with a total of 15 different object detection systems with a wide range of measurement principles, such as LiDAR, ToF camera, stereo camera, radar, and ultrasonic.

To present first measurement data and the resulting potential, an example test is used in this article. The example represents a 2D LiDAR sensor in a static test scenario. This 2D LiDAR sensor is an ODS with an adjustable SDA. If an object is detected by the sensor system within the SDA, this is passed on to the test stand controller via a digital output. The static test scenario is understood to mean that the sensor system is statically located at a fixed position and the test target is dynamically moved through the SDA of the ODS from different directions. Different parameters are evaluated, such as the reaction time or different filters for rain or fog. There is only one algorithm for the application-specific software tested. In the following examples, the detection capability of persons and the disturbance effects on the 2D LiDAR ODS in agricultural environments are presented.

### 2.1. Humanoid Test Target

**Figure 4:**
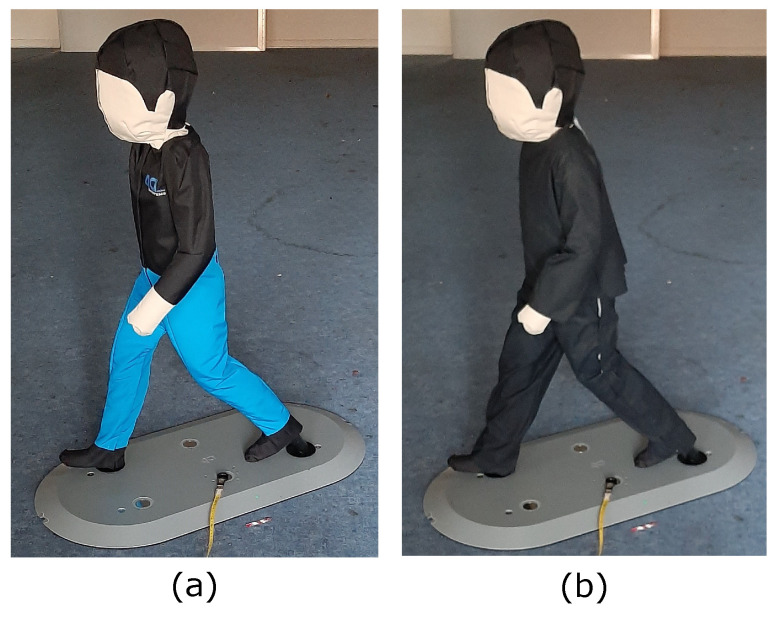
This figure presents in part (**a**) the test target defined in the standard ISO 19206-2:2018-12 [19] and in part (**b**) the modified test target with optical requirements of the standard ISO 3691-4:2020-02 [20] by the authors. Reprinted from Ref. [18].

In order to realistically test the detection capability of persons by the ODS, a modified humanoid test target is used that simulates, as much as possible, the reflectivity of a real person by an ODS [18]. The test target from the standard ISO 19206-2:2018-12 [19] is used as a base and modified with the optical requirements of the standard ISO 3691-4:2020-02 [20]. Reflection properties of other sensor measuring principles were evaluated as well. Here, a realistic reflection of the test target is already given. In Figure 4, the base test target “4activePS child (v3v3.2)” from the manufacture 4activeSystems GmbH from Traboch in Austria [21] (part (a)) and the modified test target (part (b)) are shown.

### 2.2. Test Setup

The test stand simulates different scenarios that occur during the operation of a mobile machine. In order to simulate the varied conditions and scenarios that arise during the use of a mobile machine, several scenarios are tested during each measurement. Subsequently, all scenarios are evaluated individually and compared with each other. The detailed test scenarios are shown in the authors publication [14]. The novel test method can be used in different domains of mobile machine with different speeds or test objects. Based on the parameters of the autonomous feeding mixer, the test stand moves sensors at a speed of up to 2 $\frac{m}{s}$ that arise in an agricultural operation. Movements of the humanoid test target are simulated with a speed of up to 2.3 $\frac{m}{s}$. An important focus is the entry of the test object into the SDA and the associated determination of the time of earliest detection by the ODS. Therefore, the SDA of each ODS is set to be smaller than the possible test area. This allows the test target to enter the SDA of the ODS from all sides, as in the occurrence of a real case.

The aim of the new test method is to investigate the detection capability of sensor systems under different environmental conditions. For this purpose, the detection capability of the ODS is checked at a variety of positions inside and outside the set SDA. The position of the test target and the ODS are stored in the database with a time stamp every six milliseconds in a coordinate system. For the positions, the parameters of the abscissa axis and the ordinate axis are used. This information is supplemented with the status of the ODS in the recorded position and the same time stamp. At this point, the status means the information as to whether the ODS reports a detection or not inside the SDA and whether it will be represented as a one or a zero in the output.

A test run consists of various test scenarios. The test stand tests 24 h a day, 365 days a year. A test run is triggered on the basis of specific triggers. Time-based triggers are defined that allow the test stand to start the fixed test run every three hours. In addition, there are environment-dependent triggers. If the test stand is in certain environmental conditions, such as rain or fog, a test run is also started. For further information on the measurement of the environmental conditions, see Section 2.3.

It is important for a test stand to make the tests reproducible. Therefore, each characteristic of a measurement is given a class and stored inside a database. In this way, the changes made to the test stand, the ODS, the scenarios, or the test targets are documented and subsequently traceable. It is irrelevant whether these changes were made to the hardware, ODS parameters, or the evaluation software (e.g., artificial intelligence). The different setups in the project “Agro-Saftey” are shown in the following Table 1.

As described in Table 1, the test target is the modified humanoid test target. There are two different setups for test scenarios. There are 15 sensors mounted on the sensor carrier, which work with six different measurement principles. Depending on the ODS, different parameters and software are tested. For other applications, the setup table can be modified.

The setup table of measurement example with the 2D LiDAR sensor system is shown in the following Table 2. For this sensor test, a modified test target as a simulation of a person (s. Section 2.1) and a static test scenario are used. As mentioned before, a 2D LiDAR sensor system is tested with no active filter and a defined reaction time deployed. Only one detection algorithm is used. These fixed setups are defined by the classification as the test setup.

### 2.3. Environment Measurements

In contrast to the test setups, the environmental conditions cannot be influenced. However, the influence of these environmental conditions on the ODS and whether it exists at all is a key test feature of the test stand. The outdoor position is a decisive advantage of the test stand. This means that measurements are carried out on the test stand in the weather combinations that actually occur. This is a big difference to test stands in climatic chambers [12]. By simulating weather conditions in such climatic chambers, individual weather conditions can be tested, but the combination of several weather conditions is not tested realistically. Because the test stand is located in the open field, e.g., in the agricultural environment, it is ensured that the ODS are tested in natural environmental conditions and in the operating environment of a mobile machine. These environmental conditions are recorded, classified, and sorted for each measurement. The procedure is explained below.

As the authors described in [14], a weather station is set up next to the test stand. The station measures the weather conditions during each test run and stores them in the database. It consists of the Vantage Pro 2 6163 EU weather station from the manufacturer Davis Instruments and the VISIC620 visibility meter from SICK AG. It stores all relevant values of the environment up to particles in the environment. This makes it possible to record environmental conditions, such as fog, morning dew, or even dust. This article presents the first measurement results and the resulting potential of the measurement data. For a better overview, the evaluation is limited to the following environmental conditions, which are described in Table 3:

The environmental conditions are classified individually. The classes offer the advantage that measurements with similar environmental conditions can be summarized and compared. Likewise, a defined class describes a specific environmental condition. Thus, the environmental condition during a measurement can be described using abstract classes. It is not in the authors’ interest to create new definitions for weather classes but to use generally applicable classes. Therefore, the classifications of the World Meteorological Organization are used [22]. This gives the possibility to stick the wind values to the Beaufort scale and to fog the visibility values given in Table 4. This allows for the presence of fog or dust to be automatically detected and classified into one of eight classes. The upper limit of these visibility classes is justified by the visibility meter used.

With the classifications which are made for further environmental conditions, it is referred to scales whose intention is to scale subjective perception of influences of the weather. This does not affect the meaningfulness of the units in which measurements were made, so that they were applied. In Table 5, five selected environmental conditions which should be examined in the project “Agro-Safety” are shown. The upper limits of the individual values result from the limits of the weather station used.

The environment table is supposed to be dynamic and expandable analogous to the setup table in order to test the possible influences on the ODS. An example for this could be the addition of the angle of inclination of the sun to the ODS in horizontal and vertical axis. Analogous to the test setup table, an environment table is created for each measurement. This table lists each of the measured weather conditions for a test setup with its values and stores it in the database. Subsequently, these values are divided into a class and indexed according to the strength of the measured weather condition. The environment table shows an environmental situation, resulting from the combination of all individually measured environmental condition classes that were simultaneously present during a measurement. Thus, for each individual measurement there is a table of the environmental situation, which describes the measured environmental conditions separately on the basis of individual environmental condition classes. Measurements with the same environmental situations or with the same specific environmental condition class can be filtered out during data evaluation. In Table 6, one measured environment is shown, which was measured while testing the example test setup (s. Section 2.2).

### 2.4. Point Clouds

**Figure 5:**
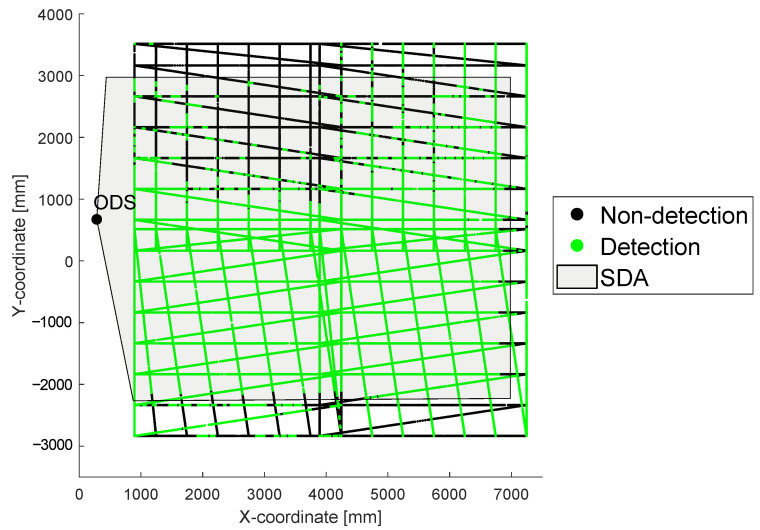
A point cloud resulting from test example with a 2D LiDAR sensor on the test stand and the example environment are shown in this graph.

To create a detection area of an ODS in a real environment, a two-dimensional point cloud is created resulting from the relative coordinates of the sensor carrier and the test target during a test scenario. Each point in the created point cloud is marked with the status of the ODS. If the ODS detects an object inside the set SDA at that moment, the point is colored in green and if the ODS detect no object, the point is colored black. The measured point cloud from the setup Table 2 and the example environment Table 6 is shown in Figure 5.

On the left side next to the point cloud, the ODS in Figure 5 is drawn. The set SDA in the ODS is shown as the gray area. The tested relative positions of the test target to the ODS are shown by green and black dots. A green dot means that when the test target was at this position, the ODS detected an object in its SDA. In contrast, a black dot means that when the test target was at this position, the ODS did not detect an object in its SDA. Due to the high resolution of the measurement, a string of measurement points can act as a line.

## 3. Data Interpretation

In this section, examples of interpretation possibilities of the measurement data from the test method “REDA” are presented. Again, there is a never-ending variety of possibilities, so that the following sections are solutions that can be arbitrarily extended or adapted for the application case.

### 3.1. Creating a REDA

**Figure 6:**
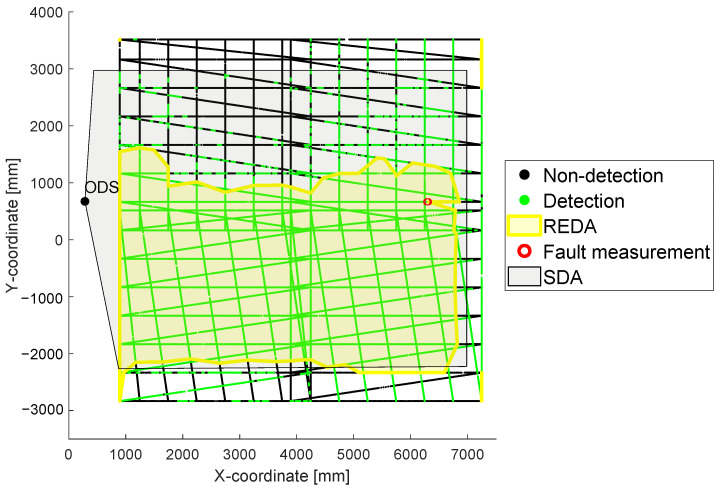
The resulting REDA area in relation to the SDA is shown in this figure for the point cloud shown in Figure 5.

For the interpretation of the measurement data, the first focus is set to the created point cloud in Section 2.4. The point cloud describes whether the ODS detected an object within its SDA with respect to the relative position of the test target to the ODS. Ideally, the SDA should be formed in the REDA with green points. For this reason, a surface area is formed from all contiguous green points. To determine this area automatically, an algorithm is developed which forms areas from all detections and areas from all non-detections. For this purpose, an algorithm is used to form a bounding envelope around the detected (green) points [23]. Subsequently, the area of all non-detections is subtracted from the area of the detections. Finally, the result is a REDA. For the point cloud shown in Figure 5 from Section 2.4, the resulting REDA is shown in relation to the SDA in Figure 6.

In the resulting REDA from Figure 5, there is a non-detection within the created REDA. In principle, a REDA is only formed on the basis of detections. Such inclusions of few non-detections are called fault measurements of the sensor systems. They are detected by the algorithm and marked in red color. Thus, detection gaps are automatically identified and the measurement is marked as erroneous for further interpretation.

**Figure 7:**
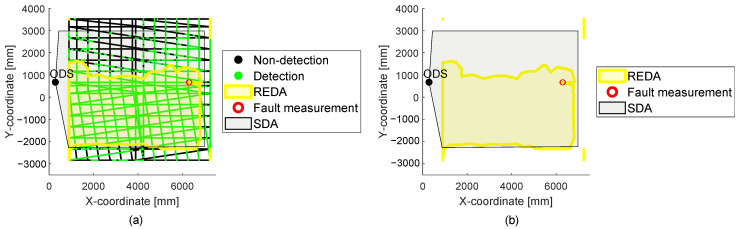
In part (**a**) the visualization of the same data from Figure 5 is shown. In part (**b**), the same measurement is shown without the point cloud.

At this point, to evaluate the measured area, it is possible to hide the point cloud. The REDA method is intended to evaluate the actual detection capability of ODS under different environmental conditions. The SDA can serve as the basis for evaluation, because it is set in the ODS and is thus expected to be the detection area. For this reason, a simplified visual comparison of the SDA and the generated REDA area is possible. In part (a) of Figure 7, the measurement with the point cloud is shown and in part (b) the same measurement without the whole point cloud is shown. Thus, a simpler and clearer manual visual observation and evaluation of the measurement is helpful. In part (a) of Figure 7, the same data from the previous figure is shown. In the visualization without the point cloud in part (b), a clearly different REDA area can be seen in comparison to the set SDA of the ODS.

### 3.2. Reda Interpretation

**Figure 8:**
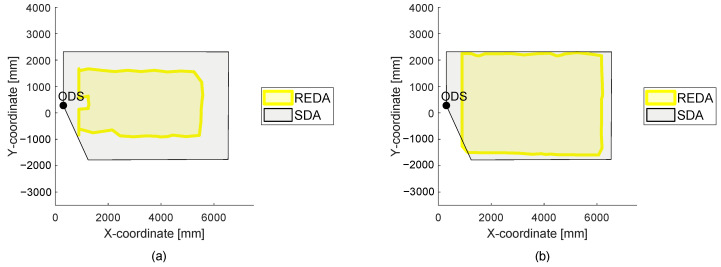
The figure presents two measurements from the 2D LiDAR sensor system example of this article. In part (**a**), a REDA of the ODS with a set reaction time of 320 ms is shown compared to a REDA of the ODS with a set reaction time of 80 ms shown in part (**b**). There are the same environmental conditions during the two measurements.

With the abstract comparable presentation of the SDA and the REDA as shown in part (b) of Figure 7, different measurement data can be interpreted by comparing the SDA and the REDA. In Figure 8, two different measured REDAs are shown with the same example as the LiDAR sensor system. The environment measurements are the same for both REDAs. The two REDAs differ only by the set parameters in the LiDAR sensor system (s. Table 1).

In part (a) of Figure 8, different reaction times are used for the example LiDAR sensor system as in part (b). The reaction time of the ODS is set to 320 ms in part (a) and is set to 80 ms in part (b). The effects of the reaction time are directly visible between the two diagrams. It is also evident from this representation that the reaction time of 80 ms results in a REDA that describes the SDA better than the REDA with a reaction time of 320 ms. Thus, for this environment measurement, the reaction time of 80 ms is better suited for the example LiDAR sensor system in the measured environment.

**Figure 9:**
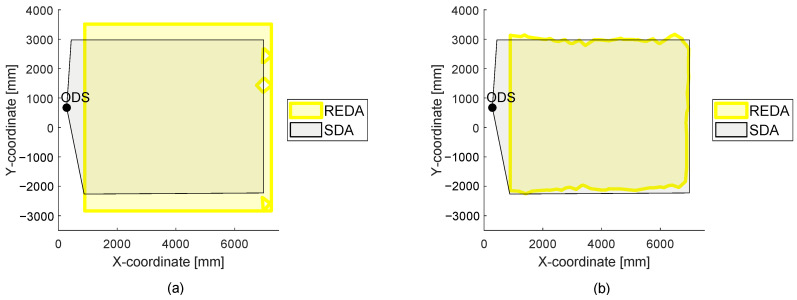
The figure presents two measurements from the 2D LiDAR sensor example of this article. In part (**a**), a REDA is shown with a visibility under 200 m due to fog. In part (**b**), a REDA is shown with no restriction of visibility due to fog. Both measurements have the same parameters and test setup.

In Figure 9, the diagrams each show two REDAs resulting from measurements with exactly the same test setup. The two measurements differ in their fog classes in the environment.

In part (a) of Figure 9, a different environmental condition is measured for the same test setup as in part (b). The difference between the diagrams is the fog class and thus the existing visibility at these measurements. The effects of the influences from the environmental conditions are directly visible between the two diagrams. In part (a), the visibility is under 200m and in the right diagram there is no restriction of visibility due to fog. In part (a), the REDA is significantly enlarged compared to the SDA and in part (b), the REDA describes the SDA much better. From this, it is concluded that the ODS during the measurement with fog in the left diagram also detected disturbances in addition to the test target and in part (b) the test target was cleanly detected. Thus, a fog with a visibility under 200m can be proven to be an influence of the ODS.

### 3.3. Detection Scores

In Section 1.1, the REDA approach is explained and the sensor specific detection area (SDA) is shown as a gray area in Figure 2. As described before, the SDA is individual adjustable in each ODS. If there is an object inside this parametrized area, the ODS has to detect the object. The status of an ODS means the information as to whether an object is detected or not and will be represented as a one or a zero in the output. This information can be used to subsequently classify the stored signals of an ODS according to whether the signal was expected or unexpected. Four different variants of points are created in a point cloud:

1. **expected detection:** If the test target is within the SDA and the ODS signals a detection;
2. **unexpected detection:** If the test target is outside the SDA and the ODS signals a detection;
3. **expected non-detection:** If the test target is outside the SDA and the ODS signals no detection;
4. **unexpected non-detection:** If the test target is within the SDA and the ODS signals no detection.

**Figure 10:**
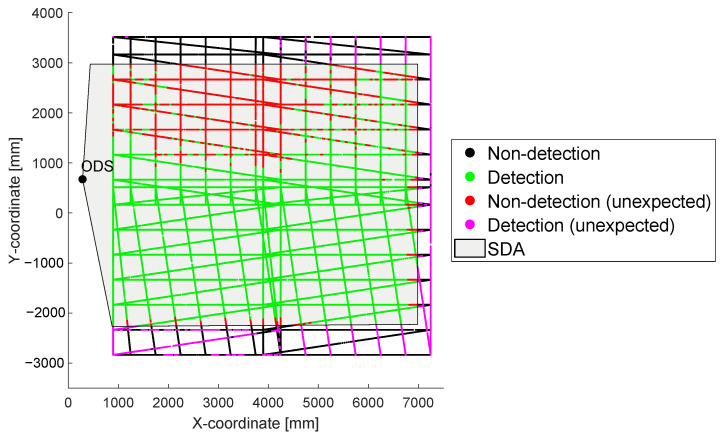
In this graph the point cloud from Figure 5 is shown with the new evaluated four variants of points.

As it is shown in Figure 10, both detections and non-detections can be subsequently evaluated; whether the status of the ODS was expected or it occurred unexpectedly depends on its status and the location of the object. The coloring of the individual points are explained in Table 7.

In Figure 10, the points of the point cloud from Section 2.4 are evaluated and marked with different colors shown in Table 7.

In order to make one measurement comparable with other measurements, scores of the point cloud are created based on the four variants of points. For this purpose, as a first step, the sum of all points inside and outside the SDA is determined.

(1) $$\sum point_{inside\;SDA}=\sum point_{non-detection(unexpected)}+\sum point_{detection(expected)}$$

(2) $$\sum point_{outside\;SDA}=\sum point_{detection(unexpected)}+\sum point_{non-detection(expected)}$$

The points totals can be used to calculate percentage shares of individual point variants. These percentages define four scores, which describe the detection capability of the ODS.

(3) $$score_{detection(expected)}=\frac{\sum point_{detection(expected)}}{\sum point_{SDA}}$$

(4) $$score_{detection(unexpected)}=\frac{\sum point_{detection(unexpected)}}{\sum point_{outsideSDA}}$$

(5) $$score_{non-detection(expected)}=\frac{\sum point_{non-detection(expected)}}{\sum point_{outsideSDA}}$$

(6) $$score_{non-detection(unexpected)}=\frac{\sum point_{non-detection(unexpected)}}{\sum point_{SDA}}$$

Applied to the measurement from Figure 10, in Table 8 the scores are calculated and listed. The sum of the percentages of the detections and the non-detections results in 100%.

### 3.4. Area Scores

Analogous to the four detection scores of the detection classification in Section 3.3, scores can also be derived for the REDA. Because the REDA represents a area, properties of an area are determined. These are called area scores in the following. There is a large number of area properties. In the following, two properties are presented as examples. Other properties are also conceivable to describe the area and make it comparable.

The first parameter used is the surface area content. The surface area of the area is given in square meters [$m^{2}$]. This parameter is of interest for the evaluation of the REDA, because the REDA is compared with the set SDA. It is checked whether the SDA is represented by the measured REDA under all environmental conditions. For example, the REDAs are compared with each other on the basis of their surface area content and with the surface area content of the set SDA. In the following Section 3.5, the possibilities of a statistical evaluation by means of the different detection and area scores from this and its preceding section are discussed. An example for the evaluation of the surface area content can be seen there.

**Figure 11:**
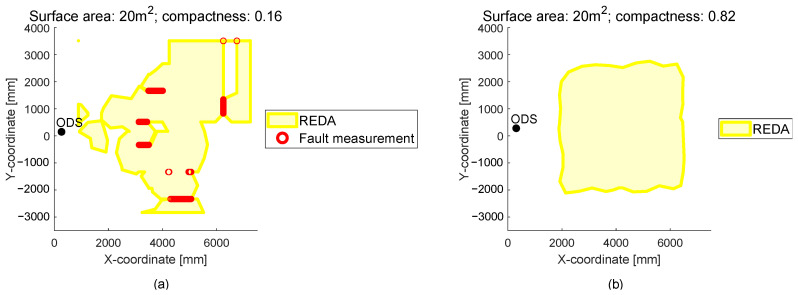
A measured REDA from an ODS with a radar sensor is shown in part (**a**). A measured REDA from an ODS with a stereo camera is shown in part (**b**). Both have a surface area of 20 $m^{2}$. Due to the difference in compactness, these two REDAs can still be distinguished.

In the following Figure 11, an example is shown, where the surface area is not a meaningful parameter for the evaluation of the REDA. For this reason, the compactness is used as another exemplary parameter.

In part (a) of Figure 11 is a measured REDA from an ODS with a RADAR sensor. The REDA has some fault measurements and consists of different areas. Together, the areas of the REDA have a surface area of 20 $m^{2}$. In part (b) of Figure 11 is a measured REDA from an ODS with a stereo camera. The REDA consists of a contiguous area without fault measurements and also of a surface area of 20 $m^{2}$. Despite the same surface area content, the two REDAs should be able to be distinguished from each other and a REDA with individual areas as in part (a) should be automatically detected and further evaluated. At this point, the compactness is helpful. The compactness gives a relation between the surface area and the perimeter of the area normalized on a circular area. For the calculation of the compactness, the Polsby-Popper score [24] is used and shown below:

(7) $$compactness=4\;\cdot \;\pi \;\cdot \;\frac{surfacearea_{REDA}}{{perimeter_{REDA}}^{2}}$$

Based on the compactness, a distinction between the REDAs of Figure 11 is possible. The REDA in part (a) has a compactness of 0.16 and the REDA in part (b) has a compactness of 0.82.

These were two exemplary parameters to evaluate and compare the area properties of REDAs. In order to determine further findings for the sensor systems, additional area parameters can be defined.

### 3.5. Statistic Evaluation

Based on the defined scores, a statistic evaluation is used which automatically evaluates the measurements and recognizes those measurements from which further knowledge about the respective ODS can be gained. Using the new test method, a large amount of data are generated. As an example, in the research project “Agro-Safety”, at least eight measurement runs with three scenarios each were recorded on 250 measurement days on the test stand. Thus, more than 6000 measurements with possible REDAs were recorded for each of the 15 sensor systems. During each of these measurements, the parameters of the test setup and the environmental conditions were recorded and classified. By determining the different scores (Section 3.3 and Section 3.4), a statistic evaluation of the measurement data is possible.

**Figure 12:**
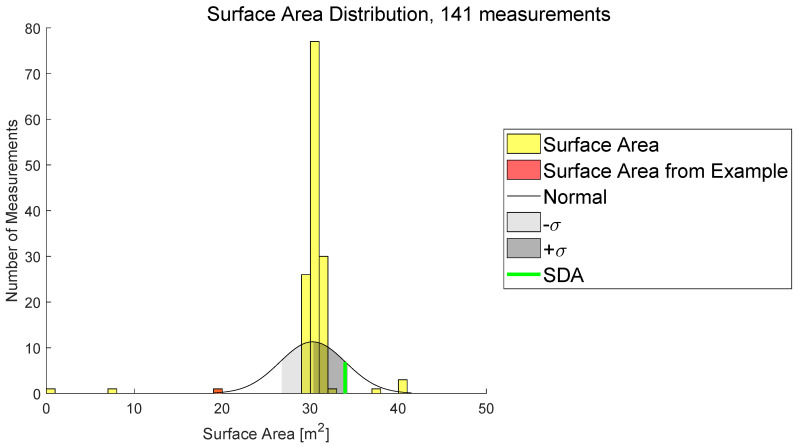
A distribution of the area of REDAs from 141 measurements of a selected test setup is shown in this histogram.

As previously described, the REDA method is intended to evaluate the actual detection capability of ODS under different environmental conditions. The SDA can serve as the basis for evaluation, because it is set in the ODS and is thus expected to be the detection area. If it is assumed that the ODS is not influenced against any environmental conditions, it should be possible to describe the SDA by the REDA for all measurements and environmental conditions. Thus, the area scores of the REDA from Section 3.4 should correspond to the scores of the SDA. At least in this case, a normal distribution (normal) of the measured surface area of the REDAs can be assumed and the surface area of the SDA should be inside the standard deviation (±1σ). Measurements outside the standard deviation of the normal distribution of area scores are filtered out and examined according to correlations of their environment class parameters. For the example of the 2D LiDAR sensor system (Section 2), the histogram of surface area scores from 141 recorded measurements with the same test setup is shown in Figure 12. Likewise, histograms can also be generated with other scores. Additionally, histograms for specific environmental conditions identify specific correlations of ODS with specific good or bad parameters or better or worse evaluation algorithms.

In Figure 12, a distribution of the surface areas of REDAs (yellow) from the test setup with the normal distribution (normal) and the standard deviation (±1σ) is shown. In red, the surface area of the REDA from Figure 6, and in green, the surface area of the set SDA in the LiDAR ODS, is marked. The SDA is inside the set standard deviation (±1σ). One reason why the SDA is not in the middle of the normal distribution is, for example, the systematic error of the measurement (e.g., reaction time). In red, the surface area of the REDA created by the measurement example of the 2D LiDAR sensor system is outside the set standard deviation. This is also evident from the optical manual inspection of the measurement in Figure 6, because the REDA turned out to be significantly smaller than the set SDA. Thus, the manual and automatic evaluation provide the same results at this point.

Next to the area scores, the same histograms can also be created using the detection scores from Section 3.3. Here, certain environmental conditions can also be filtered out which influence the ODS. Similarly, the area scores measurements outside the normal distribution of the detection score could be environmental conditions which influence the ODS. Additionally, the other way around, histograms can be created for certain environmental conditions, for example. This could identify ODS that performed particularly well or particularly poorly in this environmental condition.

At this point, the great variety of evaluation and interpretation possibilities has been pointed out once again, in terms of what this new measurement method offers with the evaluation. Influences of sensor systems are easily identified by outliers of a statistic. Any tables and scores can be expanded or modified according to the evaluation criteria of the respective test on the test stand.

### 3.6. Creating a REDAM

**Figure 13:**
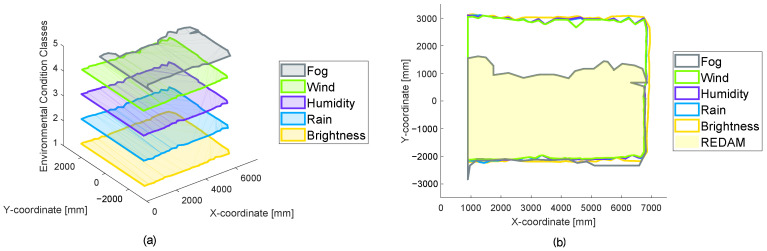
The creation of a real environment detection area matrix (REDAM) for the 2D LiDAR sensor system is shown. In part (**a**), the 3D visualization of the matrix is presented and in part (**b**) the view along the application axis with the resulting REDAM is presented.

For the LiDAR sensor system, a REDAM is created. A selection of measurements for the five selected environmental conditions of Table 6 in Section 2.3 are plotted on the application axis. The creation of a REDAM is shown in Figure 13.

The REDAM of the LiDAR sensor system is shown with REDAs for the five environmental conditions in part (a) of Figure 13. If the REDAs are viewed along the application axis (part (b)), a maximum smallest area can be created. This area is available in each REDA. This area corresponds to the detection area of the LiDAR sensor system, which results in the REDAM under all plotted environmental condition classes. Using the LiDAR sensor system as an example, it can be seen that the fog has the greatest influence on the system under the environmental conditions shown and thus reduces the final resulting detection area in the REDAM. If a REDAM is created with all measured environmental conditions, it shows the REDA resulting from the combination of individual environmental condition classes for all measured environmental condition situations.

At this point of the article, the benefit of general international definitions of environment classes is given. Up to now the necessity was not yet present, but by the protection of autonomous system such definitions become ever more necessary for the assessment of sensor systems. A comparability of sensor systems is possible at all operational areas distributed all over the world. Likewise, it is possible to fall back on classes that have already been tested. Thus, only the measurement of the occurring environmental situations with the individual environmental condition classes and the comparison with already tested environmental situations are necessary for the selection of suitable sensor systems. Classes for defined conditions on and in ground vehicle are defined, for example, in the standard IEC 60721-3-5:1997-03 [25]. Based on this standard, working condition situations relating to environmental condition classes have be defined for autonomous machines.

This is a significantly new way to test and evaluate sensor systems for outdoor use. The functionality of sensor systems is classified up to concrete environmental conditions and concrete statements are made about the limits of the functionality of the sensor systems. Thus, in the future, the diversity of environmental conditions and the associated strengths and weaknesses of the sensor systems are specified and compared with each other.

## 4. Conclusions and Outlook

In this article, measurement data and the resulting potential of the novel test method REDA by creating real environment detection areas (REDAs) published by the authors in [14] are presented. Using this method, the strengths and weaknesses of sensor systems are identified with an unprecedented level of detail, flexibility, and variance. The detection capability of sensor systems becomes measurable and comparable. Solutions for the safe detection of persons on autonomous agricultural machines are being sought, but the proof of safe functionality is very difficult to unsolve. Specific tests in singular environmental conditions, such as fog chambers [12] or rain simulation [13], only cover a small part of the versatile environmental conditions and disregard the combination of different environmental conditions. Compared to these existing test methods, the new test method REDA covers real combinations of outdoor environmental conditions. As a consequence, the new test method and the test stand enable a systematic and a depth of detail which is not reached by existing methods and technologies. Thus, it is possible to compare the detection capability for a specific object of, for example, a radar sensor with a LiDAR sensor. However, it is also possible to test and compare different parameters of the sensor system. Beyond that, different evaluation algorithms, e.g., artificial intelligence, can be compared and tested.

Thus, a test method has been developed that makes a significant contribution to the development of sensor systems and to the development and validation of autonomous systems. At the same time, it is a tool for providing evidence of the safe functionality of sensor systems in their planned operational environments. Thus, the method contributes to the compliance with the standard IEC TS 62998-1:2019-05 [10].

Likewise, all possibilities of test scenarios can be realized and compared. This method can be used on a wide variety of test stands. The use of modified use cases, modified speeds, or test targets is possible. Thus, this test method is applicable for various scenarios of environmental monitoring. The first sensor test stand realized in the “Agro-Safety” project as the first realization of this test method can also be used flexibly for various scenarios and test targets. Due to the optimal location next to a silo on a farmed area, this test stand can even be used to test special conditions of the indoor and outdoor economy of agriculture.

With the statistical evaluation, the great variety of evaluation and interpretation possibilities is shown, once again. Influences of sensor systems are easily identified by outliers of a statistic. Any tables and scores can be expanded or modified according to the evaluation criteria of the respective test on the test stand.

To present the power and versatility of the evaluation possibilities of the measurement results made possible by the new test method “REDA”, a variety of parameters of the test method are listed in Table 9 below. This table can be extended as required depending on the application. It has thus become possible to filter in the simplest way for a specific test parameter or combination and to determine the detection capability or interference sensitivity of a sensor system on the basis of the resulting REDAs.

The new test method offers completely new future possibilities for the development of sensor systems. The detection capability of a sensor system becomes comparable to other sensor systems. It has now become possible to assess the influence of environmental conditions on sensor systems detection in an unprecedented level of detail. Different parametrizations of sensor systems can be tested and compared for specific environmental conditions. This can be realized for one sensor system or compared with other sensor systems. However, the result of this new test method does not necessarily mean that different sensor systems have to be combined with each other. The best result for an application by the test method can also result from the combination of different parameters or algorithms of the same sensor system. These are completely new dimensions of comparison and development methods for the use and development of sensor systems. For the evaluation software of the sensor data, different algorithms can be tried and tested. Their effectiveness and functionality can be compared and evaluated in the simplest way.

This test method offers impressive possibilities for various applications of sensor systems. Manufacturers of autonomous machines in general and of the autonomous feeding mixer in particular can test sensor systems in the working conditions of the machine and their ability to detect objects. The humanoid test target is used to test how well persons are detected by the sensor systems. This is an essential step for the development of autonomous systems. Based on the results, a sensor system can be selected specifically for the feeding mixer.

This article has presented first results and the application of a novel test method with a unique outdoor test stand for sensor systems. In the long term, further operation will also allow measurement data to be recorded for unusual and rarely occurring weather conditions. In the future, other sensor systems, other configurations or data interpretations, such as algorithms, can be tested in the long term. Additionally, the test method can be used for different new applications. The generation of a point cloud independent of the measurement principle can be extended in the future, so that in addition to the pure information as to whether the ODS has detected an object in its SDA, the supposed position of the object detected by the ODS can also be compared with the actual position of the test object. In this way, it will additionally be checked whether the ODS has actually detected the test target in the SDA and whether its position has been correctly determined.

## Figures and Tables

**Table 1:** The different setups of the application of the novel test method “REDA” are presented in this table.

Setup	Labeling	Number of Classes
test targets	modified target (s. Section 2.1)	1
scenarios	static, dynamic	1–2
sensors	manufacturer and name	1–15
principles	radar, ultrasonic, LiDAR, camera, …	1–6
parameters	filter, reaction rate, …	n
software	algorithm 1, algorithm 2, …	n

**Table 2:** Setup table for the test example with a 2D LiDAR sensor.

Setup	Labeling	Classification
test target	modified target (s. Section 2.1)	1
scenario	static	1
sensor	example	7
principle	2D LiDAR sensor	1
parameters	no fog and rain filter activated	1
software	algorithm 1	1

**Table 3:** Overview of the selected environmental conditions in the research project “Agro-Safety”, measured by the weather station next to the test stand.

Environmental Condition	Description	Unit
rain	rainfall in millimeters per hour	$\frac{\mathrm{mm}}{h}$
fog	visibility in meters	m
wind	wind speed in kilometers per hour	$\frac{\mathrm{km}}{h}$
brightness	brightness in watts per square meter	$\frac{W}{m^{2}}$
humidity	relative humidity in percent	%

**Table 4:** Exemplary classification of visibility according to [22], which is used to measure the influences of dust and fog.

Classification	Visibility in Meters
1	10,000–16,000
2	4000–10,000
3	2000–4000
4	1000–2000
5	500–1000
6	200–500
7	50–200
8	Less than 50

**Table 5:** Overview of the classifications of five selected environmental conditions.

Environmental Condition	Measurement Range	Number of Classes
rain	0–500 $\frac{\mathrm{mm}}{h}$	7
fog	0–16,000 m	8
wind	0–1000 $\frac{\mathrm{km}}{h}$	13
brightness	0–1000 $\frac{W}{m^{2}}$	5
humidity	0–100%	3

**Table 6:** Environment table for the test example with a 2D LiDAR sensor.

Environmental Condition	Value	Classification
rain	0 $\frac{\mathrm{mm}}{h}$	1
fog	888 m	5
wind	0 $\frac{\mathrm{km}}{h}$	1
brightness	0 $\frac{W}{m^{2}}$	1
humidity	94%	3

**Table 7:** Tabular overview of the evaluated and unevaluated visualization of the expected and unexpected detection and non-detections.

ODS Signal	Unevaluated Visualization	Classification	Evaluated Visualization
detection	●	expected	●
detection	●	unexpected	●
non-detection	•	unexpected	●
non-detection	●	expected	●

**Table 8:** Tabular overview for the calculation in percent of the expected and unexpected detection and non-detections with numerical example for the measurement in Figure 10.

Plot	Score	Percentage
●	detection (expected)	78.2%
●	detection (unexpected)	21.8%
●	non-detection (expected)	74.3%
●	non-detection (unexpected)	25.7%

**Table 9:** This table represents the flexibility and variability of the new test method by a variety of test parameters.

Categories of the Test Method	Parameters	
**test stand**	time stamp	position test target
	speed	position sensor system
	acceleration	direction of movements
**sensor system**	measurement principle	parameter
	functionality	algorithm
**environment**	temperature	sun position
	rain	wind
	fog	brightness
	snow	humidity
**data interpretation**	surface area	compactness
	expected detections	expected non-detections
	unexpected detections	unexpected non-detections

## Abbreviations

The following abbreviations are used in this manuscript:

BMBF	German Federal Ministry of Education and Research
IEC	International Electrotechnical Commission
ISO	International Organization of Standardization
LiDAR	Light Detection And Ranging
ODS	Object Detection System
PAS	Publicly Available Specification
RCS	Radar Cross-Section
REDA	Real Environment Detection Area
REDAM	Real Environment Detection Area Matrix
SDA	Specified Detection Area
ToF camera	Time-of-Flight camera
TS	Technical Specification
TÜV	German Technical Inspection Agency
